# **Argon plasma coagulation is not effective in every vascular lesion**

**Published:** 2016

**Authors:** Himadri Chauhan, Sherly Mathews, Kamran Rostami

**Affiliations:** 1 *Department of Gastroenterology Milton Keynes University Hospital, United Kingdom*; 2 *Department of Pathology, Milton Keynes University Hospital, United Kingdom *

**Keywords:** Gastrointestinal (GI) bleed, GVE, HHT, angiodysplasia, APC, Argon plasma coagulation

## Question

**Figure 1:**
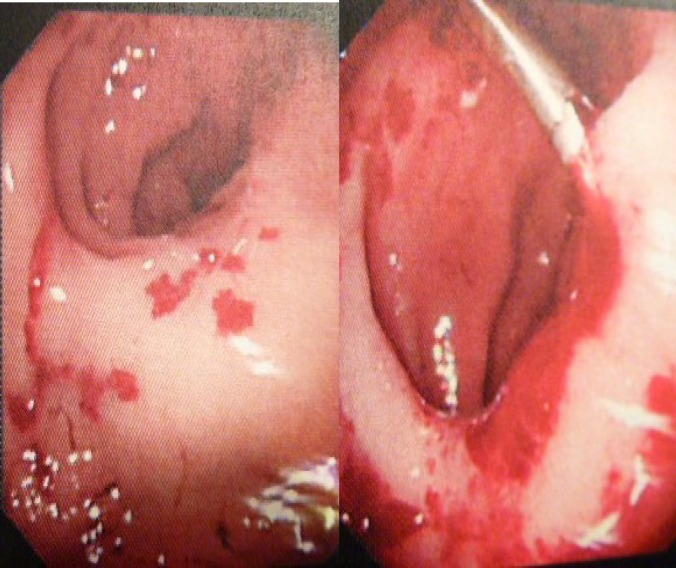
Endoscopy feature of patient A

**Patient A** is a 72 year old female referred for urgent upper intestinal endoscopy for a low Hb of 6.0 gm/dL, and a history of iron deficiency anaemia for several years. She received several blood transfusions over a period of nearly 10 years. She gives history of melaena and frequent episodes of epistaxis (figure 1).

**Patient B** is a 98 year old male, referred for urgent outpatient upper gastrointestinal endoscopy because of a low haemoglobin of 6.0 gm/dL and current complaints of haemetemesis and malaena. He had received several transfusions of blood over the past few months. This patient has no history of any stigmata of liver disease.

**Figure 2:**
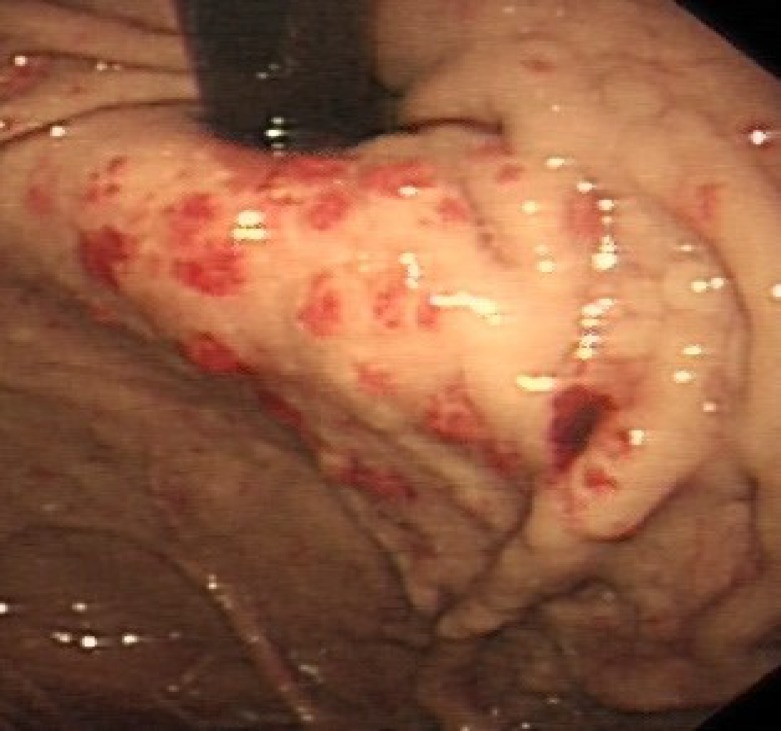
Endoscopy feature of patient B

Argon Plasma Coagulation is the safe and accepted first line of treatment in some gastrointestinal vascular disorders like the ones found in Patient B. However this treatment was not only unhelpful in Patient A, but it increased the intensity of bleeding (figure 2).

**Question 1- What is the diagnosis for Patient A and Patient B?**

**Question 2- What is the immediate and long term management for these patients?**

## Answer

**Patient A**

Patient A has hereditary haemorrhagic telangiectasia (HHT). Hereditary haemorrhagic telangiectasia (HHT), also known as Osler-Weber-Rendu disease, is a autosomal dominant hereditary disease of the fibrovascular tissue (1). It is thought that the abnormal vessels in HHT develop because of aberrant TGF signalling at some stage during vascular development and homeostasis due to the mutations of HHT associated genes (2). Telengiectasias and arteriovenous malformations in HHT are thought to arise because of changes in angiogenesis (3), during the development of blood vessels out of existing ones. The development of new blood vessel requires the activation and migration of various types of cells, chiefly endothelium, smooth muscle and pericytes. The exact mechanism by which the HHT mutations influence this process is not yet clear, but it is likely that they disrupt a balance between pro and anti angiogenic signals in blood vessels. The wall of telangiectasia is unusually friable, which explains the tendency of these lesions to bleed.

The main characteristics of the disease are abnormal capillary connections between arterioles and venules of the skin and mucosa which present as telengiectasias, which are the characteristic lesion of HHT. It arises from a dilated post capillary venule that enlarges and fuses with an arteriole bypassing the capillary system and resulting in an arteriovenous communication (4). It can present as discreet arteriovenous malformations (AVMs) or as multiple diffuse telangiectasias or aneurysms. Telengiectasias are not merely a cosmetic problem, but may cause haemorrhages in >27% of patients, mostly as haemorrhages from nose, tongue, fingers, skin of the supraclavicular fossa and can worsen the quality of life. 

The clinical manifestations of HHT are known to be variable and age dependent (1). Epistaxis is the first manifestation and the most common symptom present in more than 90% of cases (5). Patients may also have a variety of serious complications due to vascular involvement of internal organs, such as the gastrointestinal tract (20%) (6). GI endoscopy reveals visible patches of telangiectasia, including the stomach, duodenum and colon. Hepatic AVMs (<30%) (7), Pulmonary AVMs (30%) (8), and Cerebral AVMs (10%) (9). The lung and brain lesions may have silent lesions resulting in sudden morbidity or death. Pulmonary AVMs (8), can result in hypoxaemia, due to right to left shunting of blood. Also, the absence of filtering capillary beds allow emboli to reach the systemic circulation, which may cause cerebral abscess and stroke. Cerebral AVMs can lead to headaches, migraines, brain abscess, seizures, stroke, transient ischaemic attacks (TIA) and can also lead to intracerebral and subarachnoid haemorrhage (9). 

About 20% of the HHT patients are affected by symptomatic digestive tract lesions, (6), although a higher percentage have lesions that do not cause symptoms. These lesions may bleed intermittently, which may be significant enough to be noticed in the form of haemetemesis or melaena, but eventually lead to iron depletion and iron deficiency anaemia. (10).

Mild bleeding and resulting anaemia is treated with iron supplements. There is evidence supporting a role for a variety of drugs including hormone replacement therapy, octreotide, thalidomide, and steroids (11) to reduce bleeding and anaemia. Review of other therapeutic options in the literature has revealed the use of tranexemic acid (12) which is an antifibrinolytic drug that blocks the binding of plasminogen and plasmin to fibrin, thereby preventing dissolution of the hemostatic plug. Tranexemic acid is also a direct albeit weak, plasmin inhibitor. In a meta-analysis of 6 studies involving 1267 patients with acute GI bleeds, treatment with antifibrinolytic was associated with 20%-30% decrease in the rate of re-bleeding (12). Corticosteroids might be of some value, particularly in those patients who are not fit for surgery. The precise mechanism by which steroids control bleeding is unclear. It may involve the augmentation of vascular sensitivity to vasoconstrictive agents, such as vasopressin, or improvement in the integrity of the vascular endothelial lining (13). There is a report into treating HHT patient with thalidomide and bevacizumab, whereby thalidomide has been reported to regulate the expression of components of signalling pathways involved in angiogenesis, such as the VEGF pathway (14). It has also been shown to target endothelial tip cells of immature vessels. Van Custem et al, (1990) (15) reported in their frequently quoted randomized, cross-over trial, the successful treatment of chronic bleeding from gastrointestinal malformations with the use of an oestrogen-progesterone combination. There have also been solitary reports of the successful use of alpha-interferon (16), calcitonin (17) and the serotonin antagonist, cyproheptadine (18).

**Table 1:** Vascular lesion endoscopy, histology and treatment options

Vascular lesions	Histology	Endoscopy	Treatment
HHT	Arteriovenous fistula, multiple dilated vascular spaces, Loss of muscularis layer and disturbance in elastic lamina of the vessel walls.	Crusty patches of 2-10mm telangiectasia, occasionally covering a larger surface, variable distribution, more common in isolation	-AVMs pre-emptively treated by embolization -Endoscopy: injecting adrenalin and endoclips
GVE	Dilated congested mucosal capillaries in the subepithelial region, intravascular fibrin, thrombi and fibromuscular hyperplasia in the lamina propia.	visible ectatic lesions mostly in aggregation, less in isolation	Endoscopy with APC is safe and effective but is rarely curative. Also, endoscopy with thermal ablation is favoured due to low side effects and low mortality.

In some studies, (19-22), severe anaemia and episodes of severe bleeding secondary to vascular lesions have been treated with endoscopic argon plasma coagulation (APC). Contrary to the literature, APC treatment was not effective and in fact, applying APC (with straight firing 2.3 mm probe, Argon gas flow of 2.0 L/min and power 20W, which is the lowest APC setting) has contributed to increasing of bleeding in our patient with HHT. Post APC, bleeding had to be stopped by injecting adrenalin and applying endoclips. Following the procedure, Patient A was admitted for monitoring haemoglobin levels, electrolytes, blood transfusion, and was commenced on Tranexamic acid IV. She was observed closely for any further bleeding. Contrary to evidence stated in literature, that APC is safe and effective as first line of treatment in cases with telengiectasia, (19) in our experience (Patient A), this modality of treatment failed to achieve the desired effect and caused even more bleeding.

There might be some confusion in current literature with regards to the role of APC in treatment of different GI vascular lesions. It is clear that APC is very effective in treating vascular ectasia, as we found in Patient B. Similarity between vascular ectasia and HHT with similar topographic and macroscopic features might trigger this confusion. However, there is substantial differences in etiology, pathogenesis, histology, endoscopic appearance (Table 1) and response to APC in these two similar conditions. Despite the lack of effectiveness of APC in HHT, long term prognosis and survival for HHT patients is favourable, providing treatable complications like AVMs are accurately diagnosed. 

Interestingly, some studies (19-22) share similar experience and state that there is not much evidence that would substantiate or refute the claim of efficacy using APC to treat the lesions caused by HHT and recognises an urgent need to conduct prospective multicentre studies to develop evidence-based management guidelines for HHT. 

**Patient B**

The gastroscopy in this patient revealed gastric vascular ectasia (GVE). The lesions did not have the classical 'watermelon' shape spreading from the antrum and they were seen homogenously in upper/lower gastric body and across the duodenum that could easily be confused with HHT especially in the acute and active bleeding phase. The clinical presentation is either chronic, low grade chronic bleeding, often leading to iron deficiency anaemia, or acute bleeding, resulting in haemetemesis or malaena. The later was the way patient B presented/ Upper GI endoscopy is the standard diagnostic test (21), although the etiology of the GVE lesions remains under debate. GI angiodysplasia and vascular ectasia induce a wide spectrum of clinical manifestations. GVE is idiopathic but some forms of GVE especially with atypical localisations are often associated with chronic renal failure, autoimmune diseases, and cirrhosis (22). Patient B had no history of liver disease and the lesions were not consistent in appearance with gastric antral vascular ectasia (GAVE) or 'water melon stomach', which is mainly localised in the antrum (22). Our patient B had multiple small vascular ectasia in the cardia, antrum and gastric upper body. At first endoscopy, the lesions were actively oozing blood and treated with a heater probe and adrenaline injection followed by APC, (with straight firing 2.3 mm probe, Argon gas flow of 2.0 L/min and power 20W, which is the lowest APC setting). He had several treatment sessions during his admission. Repeat endoscopy showed the bleeding has reduced following APC treatment and eventually stopped before discharge. 

 Argon Plasma Coagulation (APC) is a non-touch electro-coagulation technique, which uses high frequency monopolar current conducted to target tissues through ionized argon gas (21). The potential advantage of APC lies in the limited depth of penetration, which reduces the risk of perforation and the symmetrical spread of coagulation in the surrounding mucosa. Previous studies (19-21) have showed that APC therapy is safe and in the short term effective in treating acute and chronic anaemia from vascular ectasia. It reduces blood loss and need for transfusions, except for portal hypertensive gastropathy (PHG). In a study conducted by Harrera et al (2008) (21), in 29 consecutive patients with GVE and with upper-GI bleeding, the overall success of APC treatment was 89% with only 1 recurrence of bleeding during the follow-up period (mean 18 months) and a significant rise in the haematocrit values.

Another study, (Boltin, 2014), (23), looked at the long term treatment outcomes in patients with GVE treated with APC. This study concluded that APC was safe and effective for the initial management of GVE but most patients did not experience long term resolution of upper GI bleeding and anaemia.

**Conclusion and learning points**

- In contrast to HHT, vascular ectasia with variable distribution might be treated effectively in short term with APC.
- Contrary to the current literature, that advocates the role of APC in safely managing patients with HHT, APC was not only non-effective but also made the intensity of bleeding worse in this case and other cases with HHT treated by authors. Topographic similarity between vascular ectasia and HHT might contribute to some confusion and possible optimistic conclusion in current literature.

As demonstrated in our experience, endoscopists will need to be more vigilant in differentiating the similar looking vascular lesions as seen in hereditary haemorrhagic telangiectasia (HHT) and gastric vascular ectasia (GVE) to enable them to choose the optimal modality of therapy.

## References

[1] te Veldhuis EC, te Veldhuis AH, van Dijk FS, Kwee ML, van Hagen JM, Baart JA (2008). Rendu-Osler-Weber disease: update of medical and dental considerations. Oral Surg Oral Med Oral Pathol Oral Radiol, Endod.

[2] Govani F, Shovlin CL (2009). Hereditary haemorrhagic telangiectasia: a clinical and scientific review. Eur J Hum Genet.

[3] Sadick H, Naim R, Sadick M, Hormann K, Riedel F (2005). Plasma level and tissue expression of angiogenic factors in patients with hereditary haemorrhagic telangiectasia. Int J Mol Med.

[4] Shovlin CL, Guttmacher AE, Buscarini E, Faughnan ME, Hyland RH, Westermann CJ (2000). Diagnostic criteria for hereditary haemorrhagic telengiectasia (Rendu-Osler-Weber syndrome). Am J Med Genet.

[5] Pagella F, Matti E, Chu F, Pusateri A, Tinelli C, Olivieri C (2013). Plasma Coagulation is an effective treatment for hereditary haemorrhagic telangiectasia patients with severe nose bleeds. Acta Oto-Larygologica.

[6] Kjeldsen AD, Kjeldsen J (2000). Gastrointestinal bleeding in patients with hereditary haemorrhagic telangiectasia. Am J Gastroenterol.

[7] Buscarini E, Plauchu H, Garcia-Tsao G, White RI, Sabba C, Miller F (2006). Liver involvement in hereditary haemorrhagic telangiectasia (HHT). Liver Int.

[8] Faughnan ME, Lui YW, Wirth JA, White RI (2000). Diffuse pulmonary arteriovenous malformations: characteristics and prognosis. Chest.

[9] Fulbright RK, Chaloupka JC, Putman CM, Sze GK, Merriam MM, Lee GK (1998). MR of hereditary hemorrhagic telangiectasia: prevalence and spectrum of cerebrovascular malformations. AJNR Am J Neuroradiol.

[10] Dupuis-Girod S, Bailly S, Plauchu H (2010). Hereditary hemorrhagic telangiectasia (HHT): from molecular biology to patient care. J Thromb Haemost.

[11] Sellinger CP, Ang YS (2008). Gastric antral vascular ectasia (GAVE):an update on clinical presentation, pathophysiology and treatment. Digestion.

[12] Khan S, Vaishnavi A (2009). Pharmacotherapy for gastric antral vascular ectasia: dramatic response to tranexamic acid. Gastrointest Endosc.

[13] Junquera F, Feu F, Papo M, Videla S, Armengol J, Bordas JM (2001). A multicentre, randomized, clinical trial of hormonal therapy in the prevention of re-bleeding from gastrointestinal angiodysplasia. Gastroenterology.

[14] Bauditz J, Lochs H (2007). Angiogenesis and vascular malformations: antiangiogenic drugs for treatment of gastrointestinal bleeding. World J Gastroenterol.

[15] Van Custem E, Rutgerts P, van Trappen G (1990). Treatment of bleeding gastrointestinal vascular malformations with oestrogen progesterone. Lancet.

[16] Disdier P, Schleinitz N, Perreard M, Monges D, Swiader L, Gerolami A (1995). Dramatic improvement of watermelon stomach with alpha-interferon. American Journal Gastroenterol.

[17] Kishi K, Kinoshita Y, Kitajima N, Itoh T, Watanabe M, Kawanami C (1991). Two cases of gastric antral vascular ectasia- response to medical treatment. Gastroenterol Jpn.

[18] Cabral JE, Pontes JM, Toste M, Carnacho E, Leitao M, Freitas D (1991). Watermelon stomach: treatment with a serotonin antagonist. Am J Gastroenterol.

[19] Kanai M, Hamada A, Endo Y, Takeda Y, Yamakawa M, Nishikawa H (2004). Efficacy of Argon Plasma Coagulation in Nonvariceal Upper Gastro Intestinal Bleeding. Endoscopy.

[20] Sharathkumar A, Shapiro A (2008). Hereditary haemorrhagic telangiectasia. Haemophilia.

[21] Harrerra S, Bordas JM, Llach J, Gines A, Pellise M, Fernandes-Esparrach G (2008). The beneficial effects of argon plasma coagulation in the management of different types of gastric vascular ectasia lesions in patients admitted for GI haemorrhage. Gastrointest Endosc.

[22] Probst A, Scheubel R, Wienbeck M (2001). Treatment of watermelon stomach (GAVE syndrome) by means of endoscopic argon plasma coagulation (APC): long-term outcome. Gastroenterology.

[23] Boltin D, Gingold-Belfer R, Lichtenstein L, Levi Z, Niv Y (2014). Long term treatment outcome of patients with gastric vascular ectasia treated with argon plasma coagulation. Eur J Gastroenterol Hepatol.

